# Effects of *Clinacanthus nutans* leaf extract on lipopolysaccharide -induced neuroinflammation in rats: A behavioral and ^1^H NMR-based metabolomics study

**Published:** 2019

**Authors:** Amalina Ahmad Azam, Intan Safinar Ismail, Mohd Farooq Shaikh, Khozirah Shaari, Faridah Abas

**Affiliations:** 1 *Laboratory of Natural Products, Institute of Bioscience, Universiti Putra Malaysia, Serdang, Selangor, Malaysia*; 2 *Jeffrey Cheah School of Medicine and Health Sciences, Monash University Malaysia, Bandar Sunway, Subang Jaya, Selangor, Malaysia*

**Keywords:** Neuroinflammation, LPS-induced rats, Clinacanthus nutans, Behavior, Metabolomics, Response biomarkers

## Abstract

**Objective:**

This research revealed the biochemical outcomes of metabolic dysregulation in serum associated with physiological sickness behavior following lipopolysaccharide (LPS)-induced neuroinflammation in rats, and treatment with *Clinacanthus nutans* (CN). Verification of ^1^H NMR analysis of the CN aqueous extract proved the existence of bioactive phytochemical constituents’ in extract.

**Materials and Methods:**

Twenty-five rats were subjected to unilateral stereotaxic injection of 10 µL LPS (1 mg/mL), while another ten rats were injected with phosphate-buffered saline (PBS, 10 µL) as control. Then, 29 parameters of rat behavior related to sickness were tracked by a device software (SMART 3.0.1) on days 0 and 14 of CN treatment. The acquired and accumulated data were analyzed using multivariate data analysis with the SIMCA Software package (version 13, Umetrics AB; Umeå, Sweden). The pattern trends of related groups were documented using PCA and OPLS analysis.

**Results:**

A similar ameliorated correlation pattern was detected between improvement in physiological sickness behavior and anti-inflammatory biomarkers by the ^1^H NMR spectra of the sera following treatment with CN (500 and 1000 mg/kg body weight (bw)) and the control drug (dextromethorphan hydrobromide, 5 mg/kg of rats bw) in rats. Here, 21 biomarkers were detected for neuroinflammation. Treatment with the aqueous CN extract resulted in a statistically significant alteration in neuroinflammation metabolite biomarkers, including ethanol, choline, and acetate.

**Conclusion:**

This result denotes that the metabolomics approach is a reliable tool to disclose the relationship between central neuroinflammation, and systemic metabolic and physiological disturbances which could be used for future ethno-pharmacological assessments.

## Introduction

Neuroinflammation is a pathological hallmark of many neurological disorders, including Alzheimer’s disease (AD), Parkinson’s disease (PD), amyotrophic lateral sclerosis (ALS), stroke, and epilepsy (Echeverria and Zeitlin, 2012 ▶). Neuroinflammation is also associated with mental health conditions such as depression, bipolar disorder, posttraumatic stress disorder (PTSD), and schizophrenia (Echeverria and Zeitlin, 2012 ▶). In 2013, Malaysia recorded the following neurological disorders as causes of mortality: Alzheimer’s and other dementia diseases (3,498 cases), epilepsy (296 cases), Parkinson’s disease (159 cases), other neurological disorders (138 cases), and multiple sclerosis (11 cases). The annual mortality rate since 1990 is 13.8 per 100,000 people (0.014%) and 25% of healthy life lost annually (890 per 100,000 people) established that these medical conditions should be taken more seriously in populations (global-disease-burdenhealthgrove.com, 2017 ▶).

Thus, developing effective treatments for prevention and/or treatment of neuroinflammation is crucial. However, this is a very significant challenge, as the etiopathology of neurological disorders is extremely complex, and is yet to be fully revealed (WHO, 2006 ▶). As one possible approach to develop anti-neurodegenerative agents, plant-based medicines acting on multiple targets might provide better therapeutic results than those acting on a single target. *Clinacanthus nutans* Lindau (Acanthaceae) is a traditional medicinal plant, commonly known as “Sabah Snake Grass” or “Belalai Gajah”. It is native to tropical Asia, and has been used as an important traditional medicine in Malaysia, Indonesia, and Thailand (Tuntiwachwuttikul et al., 2004 ▶; Sakdarat et al., 2009 ▶). It has been used to treat skin rashes, insect and snake bites, burns, allergic reactions, as a diuretic, and against diabetes mellitus and fever (Sakdarat et al., 2009 ▶). These uses are reportedly due to its antioxidant, anti-viral, anti-inflammatory, and anti-cancer properties (Kongkaew et al., 2011 ▶; Sookmai et al., 2011 ▶; Kunsorn et al., 2013 ▶), which have attracted wide attention and aroused the investigative interests of pharmacologists. This plant is also known to have neuromodulatory properties (Lau et al., 2014 ▶), but these effects have yet to be studied in model systems. The anti-oxidant and anti-inflammatory properties of *C. nutans* make it a reasonable choice to combat neuroinflammation.

As inflammation plays a significant role in a variety of neuronal diseases, many studies have been conducted using animal models with such conditions. There is also a long history of using rats in experimental model systems in neuroscience, which provides a wealth of information in the fields of behavior and neurophysiology (López et al., 2003 ▶; Norouzi et al., 2016 ▶; Tijani et al., 2012 ▶). A rat’s brain also shows some of the most fundamental design principles which are similar to those of humans (Abbott et al., 2010 ▶), and can be successfully used to study many neurodegenerative diseases. One of the PD models is a stereotactic LPS injection into the substantia nigra, medial forebrain, or striatum which will activate the microglia within 3 days and can be sustained up to 8 weeks (Qin et al., 2007 ▶). PD, which has been associated with stress, anxiety, and depression, typically also worsens the motor function (Metz et al., 2005 ▶; Hemmerle et al., 2012 ▶).

Metabolomics has been increasingly used as a powerful tool for the identification of molecular biomarkers in many medical areas, including diagnosis of diseases or determination of their prognosis, exploring the potential mechanism of diverse diseases, and assessing the therapeutic effects of drugs (Sethi and Brietzke, 2015 ▶). Metabolomic profiles can be achieved through high-yield sample analysis using technologies such as NMR spectroscopy, GCMS, and LCMS, followed by pattern recognition statistics (Alonso et al., 2015 ▶). The combination of the NMR analysis of biological samples and the behavioral parameters of an *in vivo* experiment in rats, examining a correlation for those metabolites responsible for the anti-neuroinflammatory activity of *C. nutans* (CN), was established through multivariate data analysis, and is reported herein.

## Materials and Methods

**Chemicals and reagents**

Deuterium oxide (D_2_O, 99.9%), deuterated methanol (CD_3_OD, 99.9%), potassium dihydrogen phosphate, and deuterated sodium hydroxide were purchased from Merck (Darmstadt, Germany). 3-Trimethylsilyl propionic acid (TSP) and lipopolysaccharide (LPS) derived from *Escherichia coli* 026: B6 were obtained from Sigma Aldrich (St. Louis, USA). The normal rat chow was purchased from Specialty Feeds (Glen Forrest, Australia).

**Preparation of the extract**

*C. nutans* plants were collected from Sendayan, Negeri Sembilan (GPS coordinates: 2°38'03.4"N, 101°53'20.5"E), Malaysia in December 2015. A voucher specimen (SK2883/15) was deposited after authentication by a botanist at the herbarium of Institute of Bioscience, Universiti Putra Malaysia, Malaysia. The leaves separated from the stems were cleaned, and then air-dried under shade at room temperature (27 to 30°C) for nine days. The dried leaves were ground into a powder using a blender, and size uniformity was ensured by sieving through a stainless-steel mesh of 200mm diameter and was stored in air-tight containers at 3±2°C before further processing. The powdered leaf material was then extracted by immersion in a measured volume of solvent (1g plant in 50ml solvent) for 3 days in a container kept away from light. The extract was filtered before repeating the process twice, each time with fresh solvent. The filtrates were combined and the solvent was removed using a rotary evaporator at 40°C. The resultant crude extract of CN (CNE) was lyophilized (extraction yield; water: 30% w/w) and kept frozen until used.

**Phytochemical analysis of the aqueous CN extract**

The proton NMR identification of the metabolites in the aqueous extracts was carried out according to Khoo and colleagues, 2015. Each extract was sampled (15 mg) and transferred into a microtube and dissolved in CD_3_OD (0.375ml) as solvent and KH_2_PO_4_ buffer in D_2_O (0.375ml, pH 6.0), containing 0.1% TSP, and the supernatant (600μl) was transferred to a 5 mm NMR tube for analysis. The spectra were acquired using a 500 MHz NMR spectrometer (Varian Inova 500, Illinois, USA) at 25°C. Phase and baseline corrections, along with spectral binning, were conducted using Chenomx software (version 7.7, Alberta, Canada) for relative quantification of metabolites. To support compound identification, J-resolved experiments were carried out.

**Experimental design for** ***in vivo*** **study**

Sixty male Sprague Dawley (SD) rats of 13 weeks old (300±50g) were obtained from the Laboratory of Animal Resources, Universiti Kebangsaan Malaysia (Bangi, Malaysia). All animal experiments were conducted in an Animal Biosafety Level – 2 (ABSL - 2) housing complex located at the same place. Three rats were housed in a polycarbonate cage and maintained in an air-conditioned room at 24±2°C and acclimatized for 7 days before experimentation. The light cycle was set for 12 hours of light and 12 hours of darkness and the rats had free access to food and water *ad libitum*.

Rats were randomly divided into the following groups; Group 1: normal rats injected with phosphate-buffered saline (PBS) + water as control (N) group; Group 2: normal rats treated with aqueous CN at 500 mg/kg bw (N+500CN); Group 3: LPS-treated rats administered with water and served as control (LPS); Groups 4-6 LPS-neuroinflammed rats treated with three doses of aqueous CN at 250mg/kg bw (CN+250), 500mg/kg of bw (LPS+500CN), and 1000mg/kg of bw (LPS+1000CN), respectively; Group 7: LPS-treated rats treated with dextromethorphan (LPS+dextro). All animal handling and experimental protocols were performed in accordance with the ethics guidelines approved by Universiti Putra Malaysia Animal Ethics Committee (Approval number: UPM/IACUC/AUP189 R070/2015). The stock solutions of CNE were separately prepared using normal water as the vehicle. The respective stock solutions were prepared and administrated by oral gavage for 14 days. For dextromethorphan, a dose of 5mg/kg bw was given. All the extracts doses were preserved at 4°C and used within three days, while dextromethorphan was freshly prepared prior to use.

**Induction of neuroinflammation in rats**

The rats were fasted overnight, then anesthetized before being positioned on a stereotaxic frame. A midline incision of the scalp was made, and the vertex area was exposed. Later, a small hole was drilled according to coordinate relative to bregma: anterior-posterior (AP) = -5.5mm, lateral-medial (LM) = +1.8mm; dorsal-ventral (DV) = -8.3mm (location of substantia nigra at right side of the brain). A single intracerebroventricular (ICV) injection (10 μl) of either LPS (1μg/1μl) freshly dissolved in PBS and filtered through a 0.22 μm membrane filter, or PBS alone, was performed for each rat using a microliter syringe (Hamilton). The injection rate was consistently programmed at 3μl per minute using a Harvard Apparatus Pump 11 elite infusion syringe (Holliston, Massachusetts, USA). One week after the injection, a behavioral test was performed and the serum was procured. For serum collection, rats from all groups were fasted for 14 hours. Each serum sample was collected into a plain vacutainer tube and then, centrifuged for 10 min at 4°C, from which the collected supernatant was stored at -80°C until analysis.

**Open field test**

The open field (OF) test consisted of a square arena made of black acrylic measuring 72cm×72cm with 36 cm walls, based on a modified method from Gellert and Varga, 2016. Rats were placed into the middle of the arena and were allowed to move freely for 5 min, while their exploration activities were recorded by a video-recorder positioned 2.1m above the apparatus. The arena was cleaned with 70% ethanol for each rat. The open-field behavior test was performed in the behavior room at a constant temperature of 25-26^o^C, lit only by a 60-Watt red lamp for background lighting. The central area was delineated virtually with SMART® software, where an imaginary inner square (18×18cm) was assessed. The overall moving trace was registered in a track motion Figure, while the locomotion of the rats and the anxiety-like activities of all required parameters, such as total distance moved (cm), moving time, average speed, proportion of total time spent in the OF arena at different speeds (cm/s), and other parameters were tracked, scored, and analyzed by SMART Video Recording System (Panlab, Harvard Apparatus, Cornellà, Barcelona, Spain) software ver. 3.1.

**Statistical analysis of behavioral study**

PCA and OPLS-DA for days 0 and 14 was carried out on the normalized data (log transformation) using the *princomp* function from the stats package of the SIMCA 13.

^1^ **H NMR spectroscopic analysis of serum**

Serum samples were thawed and a sample (200μl) was mixed with PBS (400 μl, 0.1232 g of KH_2_PO_4_), containing 10 mg trimethylsilyl propionic acid sodium salt (TSP) (10mg) prepared in D_2_O (10ml) with 1.0M NaOD solution (used to adjust the pH to 7.4), in a 5mm standard NMR tube (Norell, Sigma-Aldrich, Oakville, Ontario, Canada). The NMR spectra were recorded using a 500MHz NMR spectrometer (Varian Inova 500, Urbana, Illinois, USA) at 25°C with the parameters of pulse width (PW) 21.0μs (90°) and a relaxation delay (RD) 2.0s. In serum analysis of large molecular weight samples which included proteins, both broad and narrow peaks were detected. A presaturation sequence was used first to suppress the residual water signal with low power selective irradiation. T2 measurement Carr-Purcell-Meiboom-Gill (CPMG) experiment was then performed using the following parameters: σ of 0.0004 and big σ of 0.8; relaxation delay (RD) 0.5s with 256 transients. The CPMG experiment is able to lessen the broad signals of macromolecules and diminish/reduce the intensity to achieve a better spectral baseline (Le Guennec et al., 2017 ▶). Deuterium oxide was used as an internal lock, and TSP was used as the calibration standard, for which the chemical shift was referred at δ 0.0ppm.

**Data processing and multivariate data analysis**

All of the NMR spectra were preprocessed and bucketed according to a procedure previously reported by Ahmad Azam et al. (2017) ▶, before being imported to SIMCA-P 13.0 software package (Umetrics, Tvistevägen, UMEA°, Sweden). A total of 250 integrated regions was bucketed by peak area integration binning sized of 0.04ppm of each spectrum. Mean centered and pareto scaled data were processed prior to analysis and visualization using multivariate statistical methods. Principal Component Analysis (PCA) and Orthogonal Partial Least Squares-Discriminant Analysis (OPLS-DA) were visualized with the scores plot of the two principal components (i.e. PC1 and PC2) in which each point represented an individual spectrum of a sample. The metabolites associated with the group separation, were indicated by the corresponding loading plots. The validity and significance of the model were checked using the permutation test, CV-ANOVA, misclassification Fisher probability, and R2Y/ Q2Y values as and when applicable. PLS regression model was also computed as reported by Wold (2001) ▶.

**Statistical analysis**

The pathway analysis and heat map generation were done using Metaboanalyst 3.0, (http://www.metaboanalyst.ca), (Xia et al., 2015 ▶). Univariate analysis was performed by using the integration areas data of each metabolite. Normality of data distribution was tested by Kolmogorov-Smirnov test and one-way analysis of variance (ANOVA) was done using GraphPad Prism V 7.0 (GraphPad Software Inc., San Diego, CA, USA). Tukey’s test was chosen as the *post-hoc* analysis method. A p≤0.05 was considered statistically significant, and the values were expressed as mean±SEM.

## Results

**Phytochemical analysis of CN aqueous extract**

Comparable with data reported on proton NMR of 70% ethanolic CN leaf extract by Khoo and colleagues, 2015, this study also showed the existence of a wide range of bioactive compounds particularly triterpenes: stigmasterol, lupeol, β-sitosterol, betulin; sulfur-containing glycosides: clinacoside A, A1, A2, B, and C, and C-glycosyl flavones: schaftoside, vitexin, isovitexin, orientin and isoorientin. Supplementary Figure S1 shows the proton NMR spectra of CN aqueous leaf extract, wherein all the above compounds were identified. The highest concentration with respect to peak intensity, was found for shaftoside, followed by acetate, vanillic acid, clinacoside B, butyrate, and propionate, respectively. A few other identified and reported compounds were sugars (fructose, α-glucose, β-glucose, and sucrose); amino acids (alanine, glutamine, proline, tryptophan, valine, and isoleucine); organic acids (citric acid, formic acid, fumaric acid, cis-aconitate, glutamate, and gallic acid); phenolic compounds (catechin, quercetin, quercetin 3-O-rhamnoside, gendarucin A), and other metabolites like choline, ascorbic acid, adenine, a mixture of cerebrosides, chlorogenic acid, and citraconate. The representative ^1^H NMR spectrum of CN is presented in the Supplementary Figure S1 and the assignment of peaks is presented in Supplementary Table S2.

**Table 1:** The major biomarkers of neuroinflammation induced by LPS in rats, their fold change values and the effect of treatment with CN extract, on day 14

	**Metabolites**	**Fold Change**						
		**LPS+water/** **Normal**	**LPS+dextro/** **Normal**	**LPS+500CN/** **Normal**	**LPS+1000CN/** **Normal**	**LPS+dextro/** **LPS+water**	**LPS+500CN/** ** LPS+water**	**LPS+1000CN/** ** LPS+water**
**1**	**Creatine**	-7.15**	-0.90	+0.18**	-0.35**	+6.46##	+1.28	+2.52
**2**	**Acetate**	+2.31**	-0.79	+0.39**	-0.79	-1.83#	-0.90#	+1.82#
**3**	**Ethanol**	+14.80**	-0.49*	+0.14*	-0.12**	-7.30##	+2.05##	+1.76
**4**	**Isobutyrate**	+4.08**	-0.91	-0.29**	-0.48*	-3.72##	+1.19	-1.97
**5**	**Leucine**	+2.57**	-1.06	-0.44**	-0.63**	-2.72##	+1.13	+1.62
**6**	**2-hydroxybutyrate**	-6.29**	-0.93	-0.20**	-0.34	+5.84##	+1.28	+2.15
**7**	**Isoleucine**	+21.24**	-0.65**	-0.10**	-0.15	-13.79##	+2.21	+3.14
**8**	**Glutamate**	-5.45**	+1.00	-0.23**	-0.41*	+5.47##	+1.27	+2.24
**9**	**Pyruvate**	-3.88**	+1.13	-0.29**	-0.53*	+4.38##	+1.14	+2.07
**10**	**Citrate**	-3.63**	-0.94	-0.30**	-0.52**	+3.42	+1.10	+1.90
**11**	**Succinate**	-2.74**	-0.75*	-0.37**	-0.63*	+2.05##	+1.02	+1.71
**12**	**Glucose**	-6.90**	-0.88	-0.19**	-0.36**	+6.08##	+1.34	+2.51
**13**	**Mannose**	-5.32**	-0.88	-0.22**	-0.41**	+4.67##	+1.15	+2.16
**14**	**Histamine**	-8.18**	-0.67	-0.18**	-0.26**	+5.46	+1.46	+2.10
**15**	**Formate**	+8.28**	-0.66*	-0.17**	-0.24**	-5.44##	+1.38	+2.00
**16**	**Allantoin**	-12.84**	-0.98	-0.16**	-0.23**	+12.61#	+2.07	+2.99
**17**	**3-hydroxybutyrate**	-4.36**	-0.95	-0.24**	-0.49**	+4.14##	+1.05	+2.12
**18**	**3-hydroxymandelate**	+2.55**	-0.97	-0.73	-0.88	+2.46##	+1.86	+2.23
**19**	**Lactate**	+2.10**	+1.50*	-0.49	-0.85	-3.14##	+1.04	+1.77##
**20**	**Choline**	+0.80**	+1.11	-0.86**	+1.16	-0.89	-0.69##	-0.93##
**21**	**Alanine**	-5.70**	+0.94	-0.19**	-0.38**	+5.36##	+1.06	+2.15

Positive and negative values denoted an increase and decrease, respectively.

*** p<0.0001,

** p<0.001, and

* p<0.05 show significant differences as compared to normal, and

## p<0.01 and

# p<0.05 show significant differences as compared to LPS+water.

**Table 2:** Ingenuity pathway analysis by MetaboAnalyst (MetPA)

	**Metabolism**	**Total**	**Expected**	**Hits**	**Raw p**	**-log**	**Holm adjust**	**FDR**	**Impact**
**1**	**Valine, leucine and isoleucine biosynthesis**	11	0.149	3	0.00032	8.0298	0.02605	0.01318	0.66666
**2**	**Glyoxylate and dicarboxylate metabolism**	16	0.216	2	0.01865	3.9817	1	0.30219	0.40741
**3**	**Pyruvate metabolism**	22	0.298	3	0.00276	5.8909	0.21839	0.07464	0.24337
**4**	**Histidine metabolism**	15	0.203	1	0.18593	1.6824	1	0.94126	0.22043
**5**	**Glycolysis or Gluconeogenesis**	26	0.352	4	0.00029	8.1154	0.024212	0.01318	0.12753
**6**	**Tricarboxylate cycle (TCA cycle)**	20	0.271	2	0.02859	3.5544	1	0.33091	0.1254

* FDR- False discovery rate

The combination of diverse bioactive compounds in the CN extract strengthens the potential of this medicinal plant in terms of pharmacological properties (Le et al., 2017 ▶). Acetate, butyrate, and vitexin supplementations have shown potential anti-inflammatory and neuroprotective effects, *in vivo* and *in vitro* (Reisenauer et al., 2011 ▶; Kim et al., 2007 ▶; Chen et al., 2016 ▶). In addition, the synergistic effect of phytosterols (β-sitosterol and stigmasterol) isolated from CN was able to block the secretion of T helper 2 cytokines (IL-4 and IL-10), while stigmasterol inhibited B cell lymphocyte proliferation (Le et al., 2017 ▶). Various evidences also support the anti-inflammatory potential of phenolic compounds (Ambriz-Perez et al., 2016 ▶).

**Behavior pattern-based analysis**

**Figure 1:**
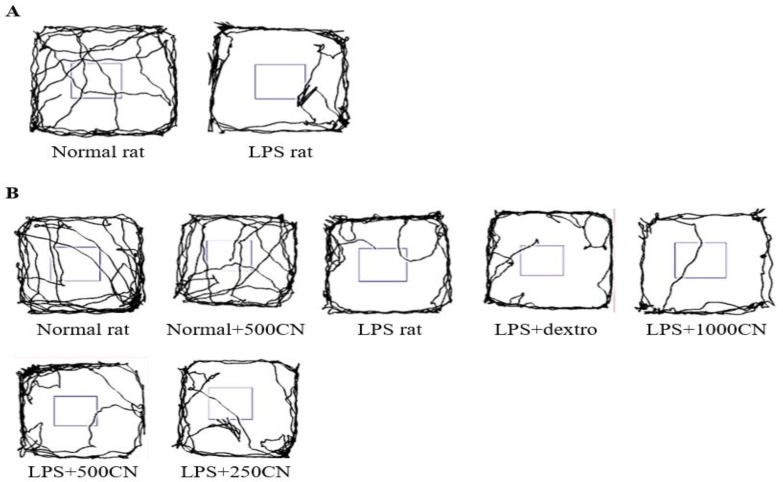
Representative traces of a rat moving in the Open Field box. The overall tracking pattern of a rat within 5 minutes on Days 0 (A) and 14 (B) with the various treatments indicated

Figure 1 shows how the SMART Video Recording System (Panlab, Harvard Apparatus) 3.0.1 software tracked the overall movement patterns of rats within 5 min in a specified box. As the rats were introduced to a new environment, the normal rats tend to repetitively cross between the zones, as they showed more lines indicating the frequency in crossing over the space. A contradictory result was shown by the LPS-induced rats, as most of them constantly moved around the peripheral zone, and rarely crossed the middle zone. The tendency to remain close to the wall was resulted from natural avert action of rodents from bright light at the centered position causing them to spend more time in protective corners in freezing state. This action has also been used as an index of anxiety whereby lessened locomotion activities of crossing in between zones is proportional to anxiety increment (Yahaya et al., 2013 ▶). Thus, it is proven that the LPS-induced rats experienced an interruption in locomotion compared to the normal rats. Nevertheless, after 14 days of treatment, no significant difference was observed between the groups under treatment.

**Figure 2:**
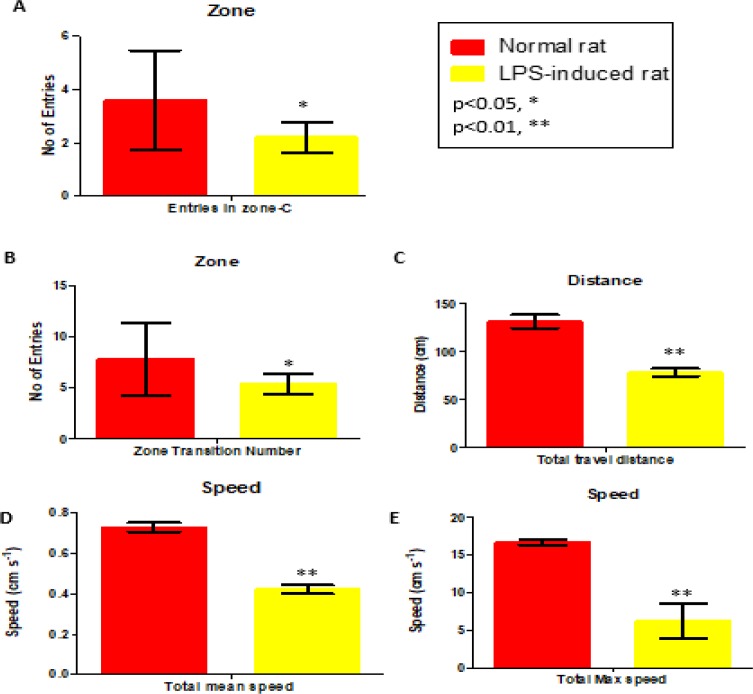
Parameters (Center-zone, distance and speed; expressed as mean±SEM) of behavior on Day 0 in comparison with the normal and LPS-induced rats. *p<0.05 and **p<0.01 show significant differences

**Figure 3:**
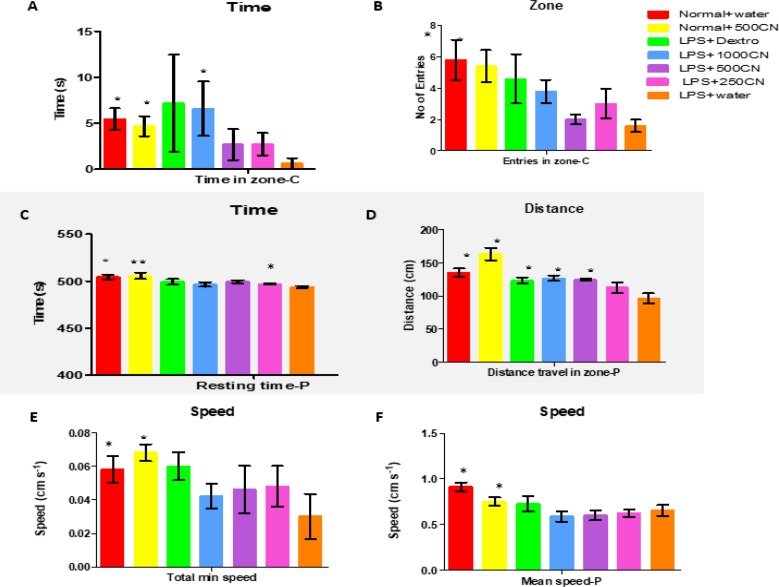
Parameters (Center-zone, distance, and speed) of group behavior on Day 14 (expressed as MEAN±SEM) compared between different groups and LPS only group. *p<0.05 and **p<0.01 indicate significant differences

Thus, 29 other parameters were used to measure the three functional categories of locomotion, exploration, and anxiety. A few of the significant parameters on days 0 and 14 of treatment are shown in Figures 2 and 3, respectively. All of the 29 parameters are shown in the Inline Supplementary Table S3 for day 0 and Table S4 for day 14.

On days 0 and 14, the LPS-induced rats exhibited significantly different behaviors in terms of the number of entries to the center area, and total travel distance in mean and maximum speed, when compared to the control group (i.e. normal rats). All of the behavioral symptoms are in line with signs of interruptions in the locomotor system which affected the exploration movement of the rats and their expression of anxiety. The behavioral symptoms matched those of patients with neuroinflammation (Lyman et al., 2014 ▶). 

Unfortunately, there was no significant difference in parameters between the LPS-treated groups with CN, drug (dextro), and LPS-only groups. Based on data presented in Table S4, only two other parameters (i.e. distance travel in the periphery and total distance travel) for the LPS+dextro, LPS+1000CN, and LPS+500CN were significantly different from the LPS only group.

**Figure 4:**
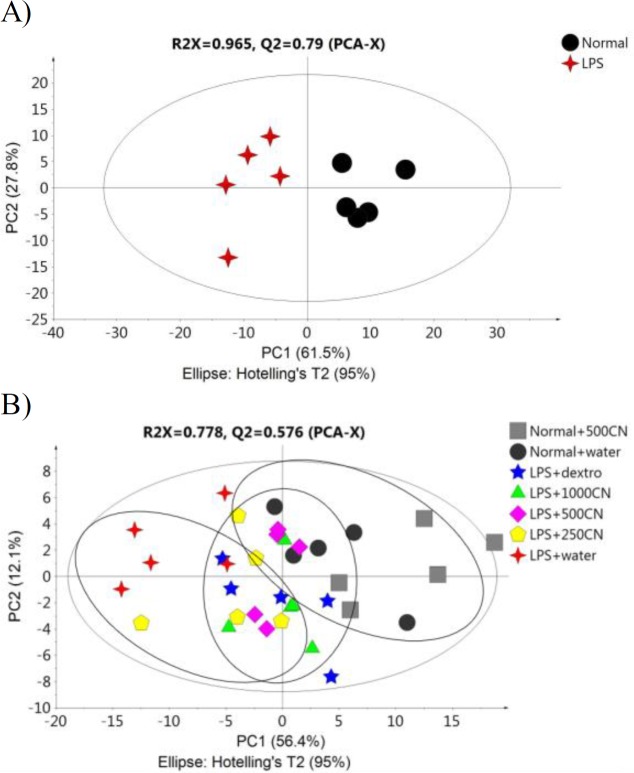
PCA score plot A (day 0) and B (day 14) concerning 29 behavioral parameters in rats

The PCA score plot (Figure 4) of the data for 29 parameters for all groups on days 0 and 14 of treatment, gives a holistic view of the symptoms and functional categories alteration induced by LPS and CN treatment. The score plot was significantly different between the two groups (Figure 4A) on day 0, which demonstrates that in all of the rats in the neuroinflammed group, neuroinflammatory condition was successfully induced. The predictive variation of component 1 corresponds to 61.5% of all variations in the data, with an R2X=0.956 and Q2=0.96. After 14-day treatment with oral aqueous CN extracts 500 mg/kg bw 1000 mg/kg bw, or with drug (dextro) (Figure 4B), the rats with neuroinflammation were observed to have shifted to the positive quadrant of component 1, approaching the normal rats. Although the medium and high doses of CN extract did not cluster with the normal group, these two doses had ameliorating effects as these groups moved farther compared to LPS-induced group.

**Figure 5:**
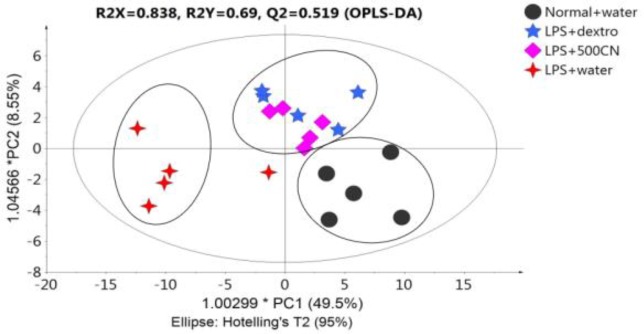
OPLS-DA score plot of four groups concerning 29 behavioral parameters in rats

**OPLS-DA analysis of the data**

Figure 5 illustrates the OPLS-DA score plot, whereby only four groups were selected in order to clearly distinguish the grouping pattern.

The model shows R2X=0.838, R2Y=0.69 and Q2X=0.519, and the predictive variation of principal component 1 is 49.5%.

**Figure 6:**
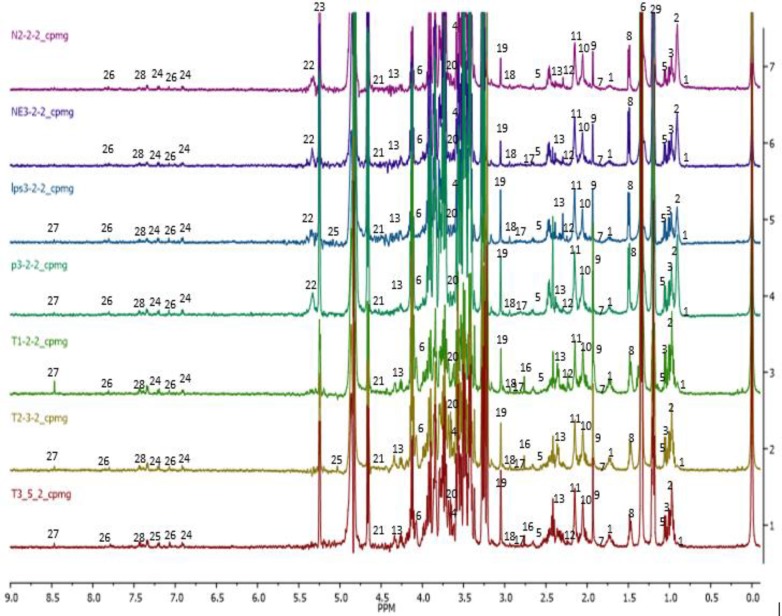
^1^H NMR of sera obtained from groups after 14 days of treatment. (1) 2-hydroxybutyrate, (2) isoleucine, (3) leucine, (4) valine, (5) isobutyrate, (6) lactate, (7) 4-hydroxybutyrate, (8) alanine, (9) acetate, (10) glutamate, (11) acetone, (12) acetoacetate, (13) 3-hydoxybutyrate, (14) succinate, (15) pyruvate, (16) citrate, (17) trimethylamine, (18) creatine, (19) choline, (20) mannose, (21) NADP+, (22) allantoin, (23) glucose, (24) tyrosine, (25) 3-hydroxymendalate, (26) histamine, (27) formate, (28) phenylalanine, and (29) ethanol

LPS-treated rats that received 500mg/kg and dextromethorphan, shared the same quadrant as that of normal rats, as referred to component 1, and LPS-treated rats without treatment were on the negative side. Therefore, CN 500mg/kg and dextromethorphan regimens showed a positive effect on neuroinflammatory symptoms in rats towards the normal conditions within 14 days of treatment.

^1^ **H NMR metabolomic analysis**

The representative ^1^H NMR of rats serum samples was obtained from the normal, neuroinflammed and CN-treated groups. The spectra denoting the pathological neuroinflammation and CNE protective effects are shown in Figure 6. Identified metabolites were labeled and assigned based on similarity search using the Chenomx NMR software database and accessible metabolomic databases, such as HMDB (http://www.hmdb.ca), METLIN (http://metlin.scripps.edu), and KEGG (http://www.kegg.jp). A total of 30 metabolites namely, 2-hydroxybutyrate, isoleucine, leucine, valine, isobutyrate, lactate, 4-hydroxybutyrate, alanine, acetate, glutamate, acetone, acetoacetate, 3-hydoxybutyrate, succinate, pyruvate, citrate, trimethylamine, creatine, choline, mannose, NADP^+^, allantoin, glucose, tyrosine, 3-hydroxymandelate, histamine, formate, phenylalanine, and ethanol were clearly identified. However, the broad water peak in the chemical shift range δ 4.65-4.8 ppm, was excluded and not used in the subsequent analyses as its broad peak tends to dominate this area of the spectrum and suppress the nearby peaks.

**Figure 7:**
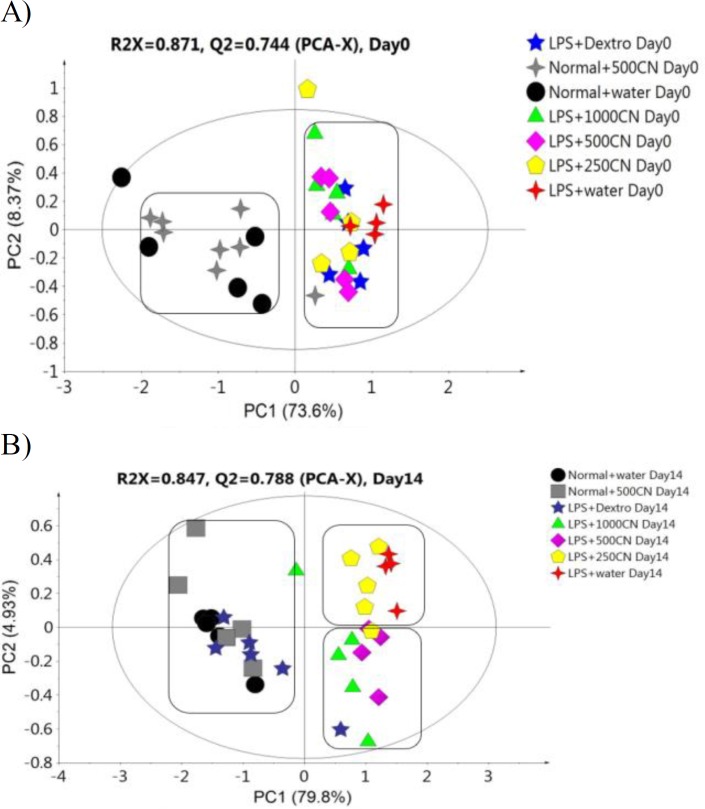
PCA score plot A (0 day) and B (14 day) based on ^1^H NMR spectra of the rat serum samples of the groups

The PCA score plot of the ^1^H NMR data of all groups on days 0 and 14 of treatment provided (Figure 7) a holistic view of the metabolite alterations induced by LPS and CN treatment. A clear distinction between the two groups was observed (Figure 7A), as on day 0, neuroinflammatory condition was induced in all rats from the neuroinflammed groups. The predictive variation of component 1 corresponds to 73.6% of all variations in the data, with an R2X=0.91 and Q2=0.86. Meanwhile, after 14 days of oral administration in Figure 7B, the neuroinflammed rats treated with CN aqueous extracts 500 and 1000mg/kg showed quite a similar trajectory pattern as those observed for neuroinflammed rats treated with dextromethorphan. Dextromethorphan-treated rats sample distribution moved closer to the normal groups, while those treated with the two doses of CN shifted away from the neuroinflammed rats that received no treatments. Although the rats subjected to both doses of CN extracts did not cluster closely to the normal group, the possible ameliorating effect of these two doses could be clearly distinguished by component 2 when comparing the trajectory changes occurred (Figures 7A and 7B). This indicates that among the treatments, the two doses of CN extract might possess an anti-neuroinflammatory activity almost comparable to that of dextromethorphan. To detail the alteration trend of variables among these three groups (two CN extract doses and dextromethorphan), the relative quantification of the possible biomarkers was carried out and values fold change with associated p-values, were tabulated in Table 1. The Table summarizes the alteration trend between LPS+dextro group and LPS+CN groups (500 and 1000mg/kg), whereby only 6 out of 21 putative biomarkers were altered in contrary to the normal or LPS+water group. Unfortunately, although the pattern of CN doses of LPS+500CN and LPS+1000CN moving away from LPS-induced group without treatment was similar to that of LPS+dextro in figure 7(B), the insignificant number of metabolites altered (6 of 21) played huge roles in discriminating these two groups of LPS+dextro and LPS+CN (500 and 1000mg/kg) as demonstrated in Figure 7B.

**Figure 8:**
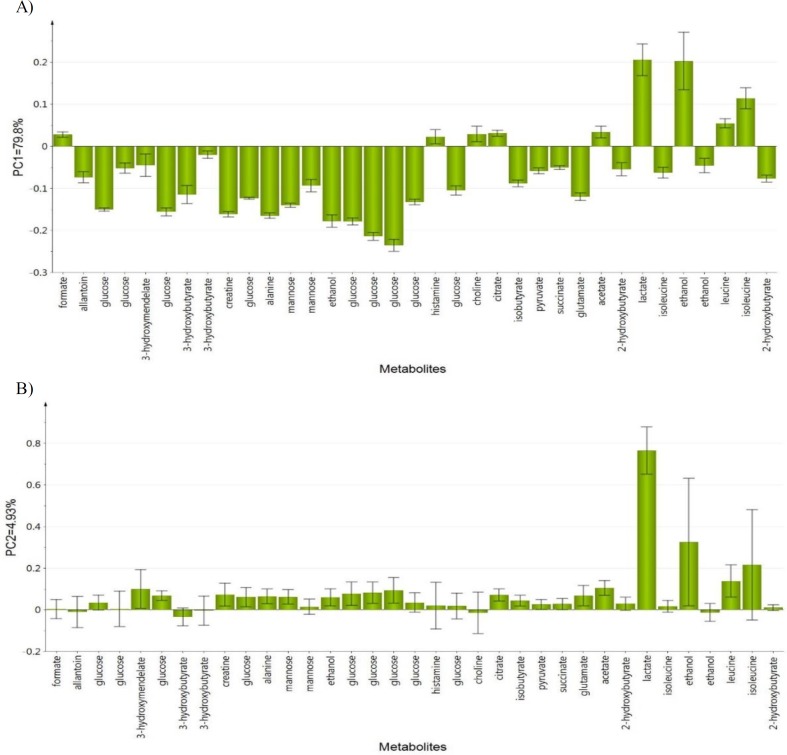
Loading column plot of PCA component 1 (A) and component 2 (B) on day 14 based on observed metabolites

Figure 8A shows the PCA loading column plots based on component 1, wherein the variables with negative PC1 belong to the classes of N, N+500CN, and LPS+dextro on day 14. This denotes that dextromethorphan has significantly altered the marker metabolites of neuroinflammation. The biomarkers of the neuroinflammed groups treated with either doses of CN also restored to those of the normal rats. The variables with positive PC1 belong to those under treatment with CN, and all of the LPS-induced rats without any treatment. The metabolites that are responsible for discriminating the higher doses (1000 and 500mg/kg bw) and lower dose (250 mg/kg bw) are shown in (Figure 8B), wherein the lower dose is on the positive side and higher doses are on the opposite.

**OPLS-DA analysis of the sera sample**

**Figure 9:**
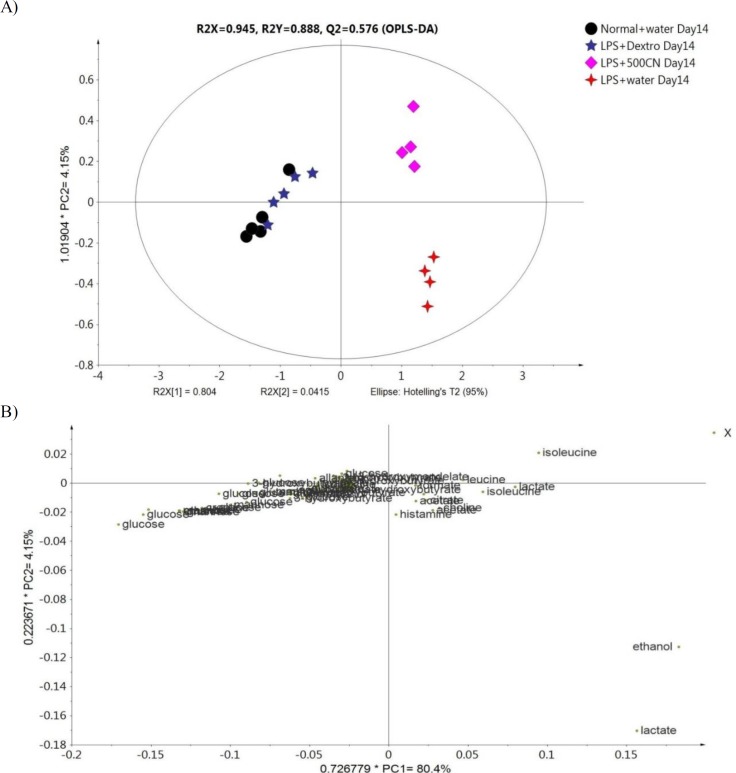
(A) OPLS-DA score plot of the 14 days group based on ^1^H NMR spectra of rat sera, and (B) the loading scatter plot of the variables

To further analyze the effect of CN on neuroinflammatory conditions, an OPLS-DA was constructed between the ^1^H NMR spectra of the sera of the N, LPS, LPS+dextro, and LPS+500CN groups. The generated OPLS-DA model was subjected to validation using misclassification prediction value, wherein a P value of Fisher probability of 2.4e^-12^ and total correction of 91.67% confirmed the validity of the model. PC1 and PC2 were described together with a total variance of 80.4% with R2Y and Q2 values of 0.888 and 0.576, respectively. As shown in Figure 9, the score plot of OPLS-DA revealed a clear discrimination along the PC1 between the normal and the LPS+dextro treated rats, with the LPS+500CN group and LPS+water group. The normal rats clustered together with the LPS-treated rats that received dextromethorphan on the left side of component 1. The clustering of the LPS-treated rats with 500 mg/kg treatment group away from the LPS group implies that the CN extract might have an ameliorating effect on neuroinflammatory conditions. 

The OPLS-DA findings are consistent with those observed in the previously mentioned PCA. The corresponding loading scatter plot for component 1, as shown in Figure 9B, reveals metabolites that contributed to the separation. The neuroinflammation biomarkers which contributed the most to the distinct clustering of the CN-treated groups are ethanol, lactate, isoleucine, leucine, and choline. The corresponding loading scatters plot based on the second component (PC2) were more important than the PC1, as the second component determines how the 500 CN extract affects the metabolites of the LPS-induced rats. The other metabolites on which CN exerted a modulating effect and derived from principal component 2, are isoleucine, leucine, and formate (Supplementary Figure S5). 

**Figure 10:**
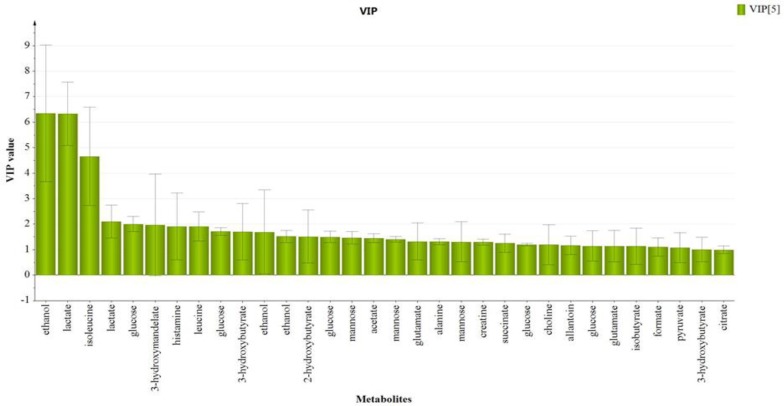
VIP scores of OPLS-DA score plot on Day 14 of treatment

The variable importance in projection (VIP) value indicates the influence of particular metabolites affecting the classification, wherein a higher value denotes a higher influence than one with lower value. The notation was taken for the variables with a VIP value>1 (Figure 10), which showed significant influence on the classification and could be selected as potential biomarkers. From the OPLS-DA model, a total of 21 endogenous metabolites were identified and characterized as potential biomarkers. The metabolites are ethanol, lactate, isoleucine, glucose, 3-hydroxymandelate, leucine, histamine, 3-hydroxybutyrate, 2-hydroxybutyrate, mannose, glutamate, alanine, creatine, choline, allantoin, isobutyrate, pyruvate, citrate, formate, acetate and succinate, respectively according to VIP score on day 14 (Supplementary Table S6). These findings are comparable with those previously reported in the metabolomics studies on neuroinflammation and several neurological illnesses (Kim et al., 2014 ▶).

**Figure 11:**
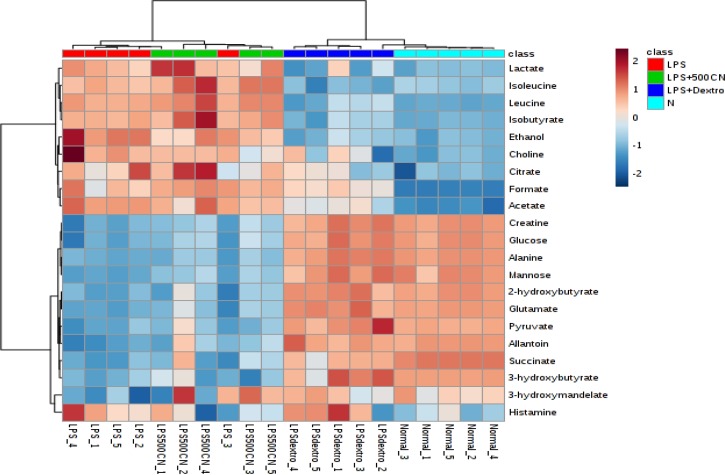
Heat map of the identified biomarkers in normal, LPS-induced, LPS+500CN, and LPS+dextromethorphan rats sera based on HCA using Ward’s minimum variance method and Euclidean distance. The concentration of each metabolite is colored based on a normalized scale of minimum −2 (dark blue) to maximum 2 (dark red)

**Figure 12:**
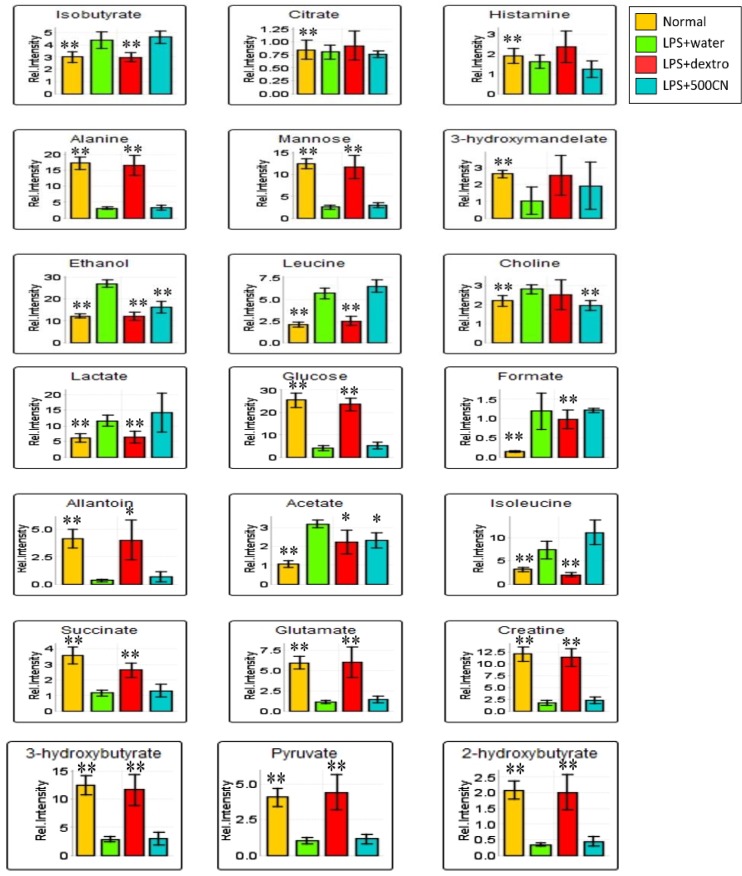
The column bar plots of the relative quantification peak intensity of the significant biomarkers found in the serum samples of normal, LPS-induced, LPS+500CN, and LPS+detxrometophan rats. Intensity of metabolites (expressed as MEAN±SEM) when comparing treatment groups and the LPS-treated rat groups. ANOVA of significant value of T-test, p<0.05, * and p<0.01, **

Supplementary data (Table S6) shows the variable region and the respective VIP score of the assigned metabolites. Through hierarchical clustering analysis (HCA) on the characteristics of the 21 biomarkers binned regions, the level of importance was normalized and Pareto-scaled before being subjected to HCA with Euclidean distance measures and Ward’s clustering algorithm. The heatmap in Figure 11 provides the details, wherein each colored box represents an average binned ^1^H NMR spectral region characterizing the metabolite importance based on the color-intensity normalized scale from minimum -2 (dark blue) to maximum 2 (dark red). The neuroinflammed group exhibited significantly higher levels of ethanol, choline, and histamine. Meanwhile, the 500CN-treated group showed a higher intensity of acetate, isoleucine, leucine, isobutyrate, citrate, and 3-hydroxymandelate, but lower levels of histamine, 3-hydroxybutyrate, and 2-hydroxybutyrate when compared with the LPS group. The dextromethorphan-treated group and the normal group displayed higher levels of other metabolites, including creatine, glucose, alanine, mannose, 2-hydroxybutyrate, glutamate, pyruvate, allantoin, succinate, 3-hydroxybutyrate, and 3-hydroxymandelate. The relative quantification of these individual biomarkers was then computed through binned data of average peak metabolites within the groups. The changes in the metabolite levels were quantitatively assessed in normal, LPS-induced, CN, and dextromethorphan treatment groups as depicted in the column plots in Figure 12.

**Figure 13:**
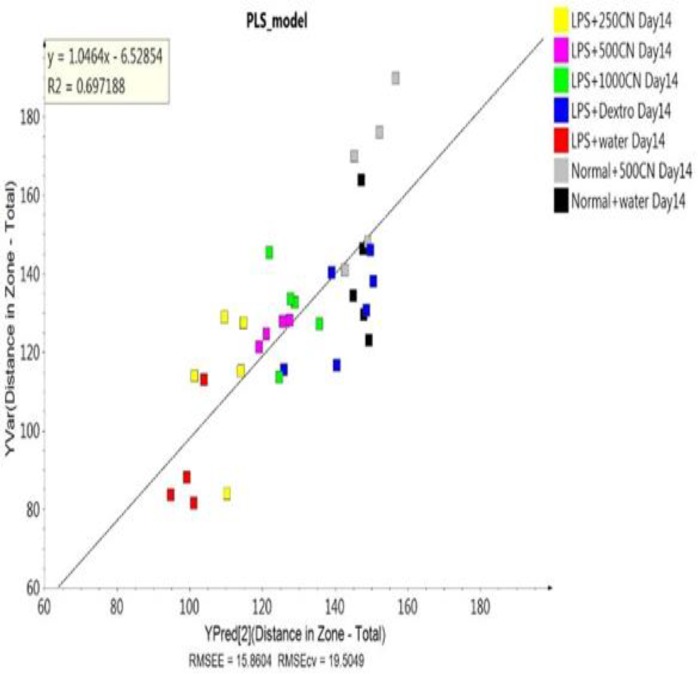
Regression model on the ^1^H NMR data of the sera for 10 behavioral parameters between the rat groups

**Figure 14:**
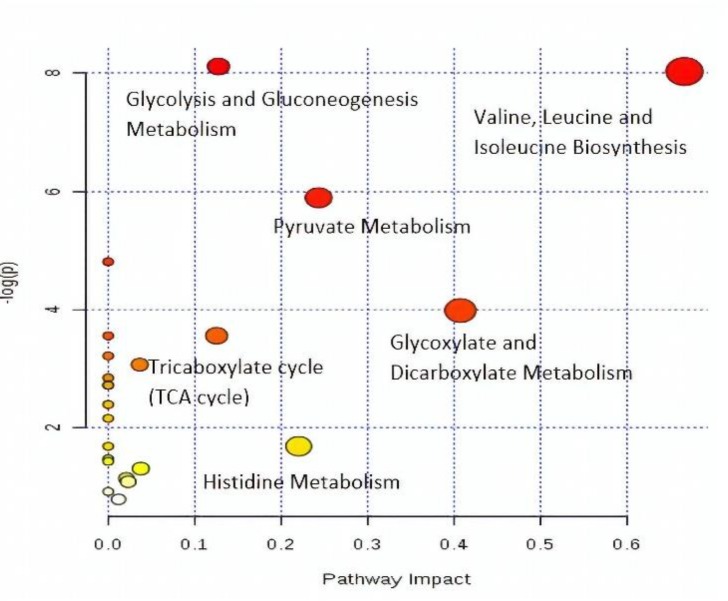
A summary of pathway analysis by MetPA

**PLS and regression model**

A validated PLS model was built to decide whether the behavioral symptoms of the rats could be hypothesized in relation to the variables obtained from PCA analysis of pattern-based behavioral observations. The model, as represented by the data, was validated based on the permutation test as shown in Supplementary Table S8. The PLS score plot and biplot also showed similarity to the validated PLS model (Supplementary Figure S9), indicating that neuroinflammation did affect both pathological hallmarks and behavioral patterns of the neuroinflammed rats. Furthermore, the regression model established that the coefficient of determination (R2=0.697) value for most of the data lies close to the linear regression line of the best fit line. It defined the data from the ^1^H NMR spectra used as X variables and the major biomarkers in behavioral pattern of 10 parameters, for which the differentiation alteration of neuroinflammation was used as Y variable (Figure 13).

**Figure 15:**
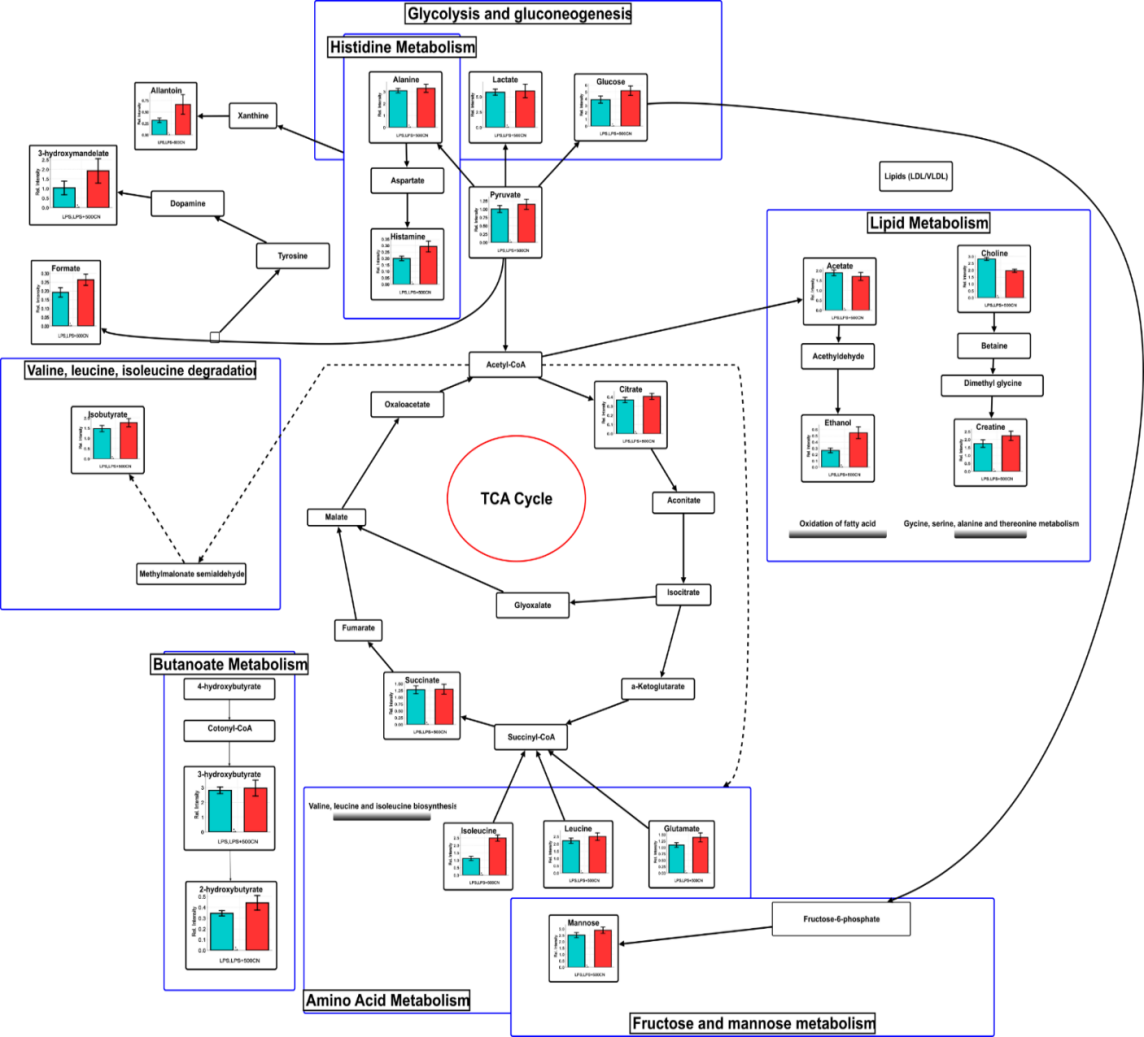
Schematic diagram of the interrelationships between the disturbed metabolic pathways identified by the ^1^H NMR serum analysis

## Discussion

**Disturbed metabolic pathways by CN in neuroinflammation**

Energy and lipid metabolisms play a vital role in the metabolic sink during inflammation and within the immune response (Purkayastha and Cai, 2013 ▶). Consequently, the identified metabolite perturbations based on the ^1^H NMR sera data between the neuroinflammatory condition and the CN- or dextromethorphan-treated rats, suggested that specific metabolic pathway alterations occurred. These metabolic pathways include glycolysis and gluconeogenesis (lactate, glucose, and pyruvate), histidine (alanine, and histamine), lipid metabolism (acetate, ethanol, choline, and creatine), TCA cycle (citrate, and succinate), amino acid metabolism (isoleucine, leucine, and glutamate), fructose and mannose metabolism, and butanoate metabolism (3-hydroxybutyrate, and 2-hydroxybutyrate). In order to systematically identify the most significant pathways which are involved in the neuroinflammatory protective mechanism of CN500, metabolic pathway analysis (MetPA) using MetaboAnalyst (www.metaboanalyst.ca/MataboAnalyst) was performed. 

Pathway impact factors are mainly used to measure the importance of metabolites in the network, and as an index to determine the most relevant pathways. Analysis of the ^1^H NMR data resulted in the identification of 20 metabolic pathways, for which the synthesis of valine, leucine, and isoleucine, with the highest pathway impact factor of 0.66, was chosen as the most significant (Table 2). Based on the set criteria for a pathway impact value of greater than 0.1, five other disturbed metabolic pathways were identified. These are glycolate and dicarboxylate metabolism, pyruvate, histidine, glycolysis or gluconeogenesis, and the TCA cycle (Figure 14). Hence, these pathways are presented as potential targeted pathways of CN treatment in a neuroinflammatory condition.

**Amino acid metabolism**

The biosynthesis of valine, isoleucine, and leucine is a part of branched-chain amino acid (BCAA) metabolism. They are important nutrients and signaling molecules, especially for the synthesis of neurotransmitters and for maintaining nitrogen balance in glutamate-glutamine cycle between astrocytes and neurons (Bixel et al., 2001 ▶; Murín et al., 2008 ▶). Various studies have suggested that increased levels of BCAAs are associated with poor metabolic health. In relation to neuroinflammation, the excessive intake of BCAAs might cause neurotoxic conditions. It was reported that the elevation of BCAAs is due to the uptake of amino acid precursors of dopamine and 5-hydroxytryptamine in a sequence response to a typical proinflammatory challenge, such as lipopolysaccharides (Crandall et al., 1983 ▶; Fernstrom, 2005 ▶). The levels of BCAA, such as leucine and isoleucine, were raised in the serum of neuroinflammed rats. Accumulation of these ketogenic amino acids acts as gluconeogenic precursors, resulting in lactate accumulation in the blood. Meanwhile, glutamate is converted into GABA, which acts as an inhibitory neurotransmitter and is recycled through the TCA cycle to synthesize glutamate (Petroff, 2002 ▶). Thus, a raised or decreased level of glutamate will create an imbalance in the glutamatergic/GABAergic level which signals the onset of the neuroinflammatory condition (El-Ansary and Al-Ayadhi, 2014 ▶). The lower level of glutamate found in the neuroinflammed rats and the CN-treated group affected the partial reversal of the elevated BCAA and glutamate. Hence, it hints at the suppression of ketogenesis or gluconeogenesis, which further supports the metabolic alterations observed under lipid metabolism through glutamate alteration of glycolate and dicarboxylate metabolism. The increased amount of amino acids in the neuroinflammed rats also suggests the perturbation of protein synthesis, which could be the reason for the observed weight loss, especially during the first three days after LPS-induction (Supplementary data, Figure S7).

**Tricarboxylic acid (TCA) cycle**

As glutamate reduction occurred in the neuroinflammatory condition, a reduction in the level of tricarboxylic acid (TCA) intermediates namely, citrate and succinate, was observed. The TCA cycle occurs in the matrix of an intracellular structure called mitochondria. The TCA cycle is a stage in cellular respiration, as it is comprised of both anabolic and catabolic biochemical pathways, which derive ATP energy from the electron transport chain (Jonckheere et al., 2012 ▶). Most of the metabolic processes in mammals, such as gluconeogenesis/glycolysis, pyruvate oxidation, lipogenesis, and amino acid biosynthesis are related to TCA cycle activities. Acetyl-CoA, produced by pyruvate in glycolysis involved in a series of reactions mediated by pyruvate dehydrogenase, which is linked with glycogen, glucose, and lactate. Consequently, a decreased level of glucose in the neuroinflammed rats affected the level of pyruvate, the product of glycolysis, while lactate was elevated. This is the scenario of hypoxia, or deficiency of oxygen (Solaini et al., 2010 ▶), which is a common feature of inflammation in PD patients (Maragakis and Rothstein, 2001 ▶). Thus, an increased level of citrate and partly succinate, suggested that CN treatment can potentially modulate the TCA cycle by up-regulating the glycolysis and down-regulating gluconeogenesis.

**Glycolysis/gluconeogenesis**

Glucose homeostasis is crucial for good health, since it handles energy production. Glycolysis and gluconeogenesis are essential metabolic mechanisms that ensure energy balance in an organism. Glucose produces the end-product of pyruvate, as it derives ATP and NADH during energy production. As pyruvate in the neuroinflammed rat declines, glycolysis activity also decreases due to the low glucose level. The pro-inflammatory cytokines stimulate insulin resistance by inhibiting insulin signal transduction (De Luca and Olefsky, 2008 ▶). Thus, deprivation of glucose will result in a reduction of pyruvate. This might contribute to mitochondrial dysfunction, as pyruvate is used as the source of energy, which in turn decreases the intermediates of TCA cycle such as succinate, citrate, and hydroxybutyrate. All of these reactions were observed in a former study, whereby the reduction of TCA intermediates in neuroinflammatory condition was shown to be due to mitochondrial dysfunction (Bradford et al., 2009 ▶).

Lactate is also an important product of glycolysis, in addition to pyruvate (Schurr and Payne, 2007 ▶), as neurons also use lactate as an energy source.49 Preference for lactate over glucose, even with oxygen availability, is reported in neuroinflammation cases (Bélanger et al., 2011 ▶). Moreover, a putative mitochondrial lactate oxidation complex, which allows entry and oxidation within mitochondria, has been described to exist in neuron neurons (Hashimoto et al., 2008 ▶). Interestingly, growing evidence suggests that neurons can use lactate as an energy source. This suggests that neurons and astrocytes may share a form of “coupled lactate metabolism”, a mechanism by which astrocytes glycolytically convert glucose to lactate, and release it into the extracellular space, where it is taken up into the neighboring neurons and utilized as a metabolic substrate for oxidative phosphorylation (Vibulsreth et al., 1987 ▶; Walz et al., 1988 ▶). More recently, oligodendrocytes were found to be vital suppliers of lactate to axons axons (Fünfschilling et al., 2012 ▶; Lee et al., 2012 ▶), through the lactate transporter MCT-1 (Lee et al., 2012 ▶). The process of oxidative phosphorylation yields 38 moles of ATP from just one mole of glucose compared to a mere two moles of ATP for every mole of glucose through glycolysis. Lactate levels can also be modulated for other reasons, such as due to food intake (Goucham and Nicolaïdis, 1999 ▶) and motor movement (Kuwabara et al., 1995 ▶).

**Glycoxylate and dicarbocylate metabolism**

The glyoxylate cycle involves the biosynthesis of carbohydrates from fatty acids or two carbon precursors which enter the acetyl-CoA pool. LPS-induction in rats by intracerebroventricular (ICV) injection delayed a reversible oxidative damage and led to neuronal loss in the targeted cells (Milatovic et al., 2003 ▶). Neuroinflammation with activated astrocytes and microglia in brain disorders, is often associated with elevated myo-inositol (Chang et al., 2013 ▶), total creatine and choline-containing compounds (Maddock and Buonocore, 2011), which indicates glial activation. The neuronal injury is detectable by the lower levels of glutamate (Hinzman et al., 2012 ▶). These process abnormalities in lipid and fatty acid metabolism cause dyslipidemia, which can be associated with increased inflammation which is well established in conditions such as atherosclerosis, cardiovascular disease, metabolic syndrome, and obesity (NCEP, 2001). The increasing level of free fatty acids eventually shifts the energy metabolism from glucose to lipids through β-oxidation of fatty acids with the aid of acetate. The increased level of acetoacetate and 3-hydroxybutyrate (3-HBT) in neuroinflammed rats indicates activation of the ketogenesis pathway, resulting in the observed mitochondrial dysfunction in neuroinflammed rats (Guzmán and Blázquez, 2004 ▶). Compared with the LPS-induced neuroinflammed rats, CN-treated rats showed a remarkable reversal in the 3-HBT level, suggesting the re-establishment of energy metabolism through glucose regulation. The increasing levels of major ketone bodies, such as 3-HBT and 2-hydroxybutyrate (2-HBT) in the CN- and dextromethorphan-treated groups compared to the LPS-treated control group, indicate that they have assisted in resolving the inflammation, as a ketogenic diet and produce a neuroprotective effect (Gasior et al., 2006 ▶). β-hydroxybutyric acid has also been found to increase brain-derived, neurotrophic factor (BNDF) levels and TrkB signaling in the hippocampus (Sleiman et al., 2016 ▶). These findings might have clinical relevance in the treatment of depression, anxiety, and cognitive impairment. 

This is the first report on the ^1^H NMR-based analysis of the serum metabolites related to the behavioral outcomes, used to evaluate the protective effects of LPS-induced neuroinflammatory condition in rats. Pattern recognition, combined with multivariate statistical analysis, suggested that fourteen days of CN oral administration at the dose of 500 mg/kg bw provided an ameliorative effect on the induced neuroinflammation. Twenty-one metabolites were identified as potential biomarkers due to their significant changes observed after 14 days of treatment. Based on these selected biomarkers, several metabolic pathways are considered modulated by CN treatment. The CN anti-neuroinflammatory activity involved the regulation of valine, leucine, isoleucine degradation, pyruvate metabolism, TCA cycle, glycolysis/gluconeogenesis, and histidine metabolism. Through this study, it was shown that a metabolomics approach is a reliable tool for ethnopharmacological assessment in traditional medicinal research.
